# Bovine mastitis prevalence and associated risk factors in dairy cows in Nyagatare District, Rwanda

**DOI:** 10.4102/jsava.v86i1.1228

**Published:** 2015-07-14

**Authors:** Blaise Iraguha, Humphrey Hamudikuwanda, Borden Mushonga

**Affiliations:** 1 Rwanda Dairy Competitiveness Program II, Kigali, Rwanda; 2 Department of Veterinary Medicine, University of Namibia, Namibia

## Abstract

In response to farmer requests after milk from their herds was rejected by processors due to poor quality, a study was carried out from April to October 2011 to determine the prevalence of subclinical mastitis, associated risk factors and causative micro-organisms. Samples were collected from 195 dairy cows on 23 randomly selected dairy farms delivering milk to Isangano, Kirebe and Nyagatare milk collection centres in Nyagatare District, Rwanda. The Draminsk^®^ Mastitis Detector was used to detect subclinical mastitis in individual cows based on milk electrical conductivity changes. Risk factors for mastitis that were evaluated included teat-end condition, cow dirtiness, breed, parity, age and stage of lactation. Relationships of these factors with mastitis status were determined using Chi-square analysis, and relative importance as causes of mastitis was assessed using logistic regression. Samples from 16 subclinical mastitis positive dairy cows were analysed to identify causative micro-organisms using Dairy Quality Control Inspection analytical kits. Subclinical mastitis prevalence was 52% across the farms. It was higher with increases in, amongst other risk factors, teat-end damage severity, cow dirtiness, and level of pure dairy breed genetics. The risk factors considered accounted for 62% of mastitis prevalence; teat-end condition alone accounted for 30%. Most of the mastitis cases (87.5%) were caused by coliform bacteria. Considering that farmers are upgrading their local Ankole cows to cross-breed dairy cows that are more susceptible to mastitis, results from this study indicate the need to dip the teats of cows in sanitisers, improve cow hygiene, and introduce mastitis prevention and control programmes.

## Introduction

In Rwanda the dairy subsector is the major player in the livestock industry (Shem 2002). National milk production increased almost three-fold from 98 567 metric tonnes in 2000 to 158 764 metric tonnes in 2008 (SNV 2009), and further increased to 445 000 metric tonnes in 2012 (Ministry of Agriculture and Animal Resources 2012). There was a glaring shortage of milk, further worsened by many setbacks including shortage of land, limited availability of feeds, unsuitable cattle breeds for high milk production, lack of funding and high prevalence of disease.

According to Bogni *et al.* (2011) mastitis is a major disease problem that blights the dairy subsector. Biffa, Debela and Beyene (2005) stated that mastitis is the most complex and costly disease of dairy cows occurring throughout the world. Mastitis causes direct economic losses in several ways, including reduction of milk yields, condemnation of milk due to bacterial or antibiotic contamination, treatment costs, higher than normal culling rates and occasionally deaths (Vaarst & Envoldsen 1997). It has been shown that mastitis reduces the quality and quantity of milk, leading to losses of margins as high as $83.37 per cow per year (Food and Agriculture Organization 2009).

This study investigated the prevalence of clinical and subclinical mastitis, the associated risk factors, and mastitis-causative organisms in dairy cows in Nyagatare District, Eastern Province, Rwanda.

## Materials and methods

### Study area

Nyagatare District is located in the Eastern Province, in the north-eastern part of Rwanda (latitude:-1°18's; longitude: 30°19’E). It is 158 km from Kigali, the capital city of Rwanda, and about 35 km from the Ugandan border (Kagitumba). It is the largest dairy district in Rwanda and has eight active milk collection centres (MCCs).

Nyagatare District is characterised by two main seasons: one long, dry season that lasts for 3–5 months; and a short rainy season that lasts 2–3 months and a second rainy season of 4–6 months. It has an annual average temperature varying between 25.3 °C and 27.7 °C, and receives an annual rainfall of 827 mm. The altitude is 1513 m above sea level. The District of Nyagatare consists of gently sloping hills separated by low granite valleys. The vegetation type is largely savannah vegetation and some gallery forestry. The major soil type is vertisols, which are rich in nutrient mineral elements but lack organic matter.

### Sample selection

Stratified random sampling was used to select three MCCs (Isangano, Kirebe and Nyagatare) from the eight active MCCs in Nyagatare District. The basis for stratification was the production systems used by Nyagatare District farmers (semi-intensive and extensive systems). The extensive system was defined as a production system where livestock are left to wander and graze during the day but are enclosed at night. The semi-intensive system was defined as a production system where cattle graze freely on pasture but receive supplementary feeds, particularly during milking, and spend the night in a kraal.

At the three individual randomly selected MCCs, eight farmers’ herds were randomly selected for study. However, at one MCC seven herds were eventually included in the study. Based on a sample size calculation (Martin, Magnani & Robert 1987, cited by Mudakangwa 2010), 195 lactating cows (65 cows at each of the three MCC) from the 23 dairy farms were included in the study. The number of cows per farm varied from 3 to 17. The cow breeds used in the study were pure-breed Friesian, Jersey, Sahiwal and Ankole and, in general, defined and undefined cross-breeds largely of the pure dairy breeds and Ankole. Breed distribution across the three MCCs was estimated to be 51% local breed (mostly Ankole), 38% cross-breeds of local and dairy breeds, and 11% pure dairy breeds.

### Data collection

Data were collected through field visits from June to August 2011. A questionnaire was used to collect basic information from farmers, and records on daily farm management were taken. Specifically the questionnaire was used to collect information including farm location and farmer contact details, cow lactation (e.g. age, parity and stage of lactation) and mastitis records.

Each cow was observed and scored for dirtiness and teat-end condition and checked for clinical mastitis visually. Samples of milk from the four quarters were tested using the milk electrical conductivity-based Draminski^®^ Mastitis Detector (see http://www.draminski.com).

Cow dirtiness was ranked based on an adopted scoring chart, 1 being clean and 4 being very dirty (Chiappini *et al.* 1994). Teat-end score was also ranked: 1 was a supple and ideal condition and 5 was the score given for keratinised and damaged teats (Animal Sciences Group 1998). After hand washing with clean tap water the operator checked udders and teats for cleanliness. Obviously dirty udders and/or teats were hand washed with clean water. After fore-stripping four to five streams of milk, the teats were dipped in 0.5% iodine solution for at least 30 seconds and then dried with disposable paper towels. After switching on the Draminski^®^ apparatus, about 15 mL of milk was stripped into a Draminski^®^ cup previously disinfected using methylated spirits, and the milk discarded after a reading appeared on the LCD panel of the Draminski^®^ apparatus as described by the Draminski^®^ Mastitis Detector manufacturer (Draminski 1989). The on/off button was then pressed.

The process was repeated for each teat, with care being taken to avoid contamination of the teats. At the end of the process the on/off button was pressed again, and the LCD displayed readings for the four teats/quarters. The readings were then recorded for each cow. Interpretation of results was based on inter-quarter variations, as described by the Draminski^®^ Mastitis Detector manufacturer (http://www.draminski.com). In between sampling of individual cows, the equipment cup and electrodes were rinsed with methylated spirits and wiped using a paper towel dipped in methylated spirits.

### Identification of mastitis-causative micro-organisms

Composite milk samples from 16 randomly selected dairy cows (out of all cows whose composite milk tested positive on the Draminski^®^ mastitis detector) were aseptically collected directly from teats into aseptic tubes and taken to the laboratory for bacteriological analysis to identify mastitis-causative micro-organisms.

Throughout the milk sample collection process each teat was first cleaned, disinfected by dipping each teat in 0.5% iodine solution and dried with a sterile paper towel. Then about 15 mL – 20 mL of milk was directly stripped from teats into sterile tubes, after which the tubes were labelled and transported on ice in a cooler box, within a period of 3–4 hours, to the Dairy Quality Assurance Laboratory (DQAL) for bacteriological examination. The Dairy Quality Control Inspection (DQCI^®^) mastitis quad plates for Staphylococcus, Streptococcus and Gram-negatives and Staphylococcus, Streptococcus and Gram-negatives chromogenic were used to identify mastitis-causative micro-organisms.

At DQAL milk samples were plated out on non-selective blood agar capable of growing the cited bacteria and using a loop (recommended for all plates, especially Staphylococcus and Streptococcus environmental plates) to spread milk from one sample vial onto each medium contained on a plate. Plates were incubated at 35 °C for 24–48 hours and the results were read within 24 and 48 hours. The methods used for micro-organism differentiation in the laboratory consisted of bacterial growth on DQCI^®^ Mastitis Media. On the media coliforms appeared pink, *Staph. aureus* showed haemolysis, whilst environmental *Streptococcus* spp. appeared brownish but with no haemolysis (http://www.dairyquality.com).

### Data analysis

Information and data from questionnaires were chronologically encoded in Microsoft Excel sheets. The Statistical Package for the Social Sciences was used to analyse the data. Descriptive statistics, Chi-square analysis and logistic regression were computed to determine mastitis prevalence, association of risk factors with mastitis prevalence, and relative importance of the risk factors on mastitis prevalence, respectively. Risk factors (e.g. teat-end condition) that had significant associations with mastitis status were included in the logistic regression model. The dependent variable of the model was cow mastitis status whilst the risk factors, including teat condition, breed, stage of lactation, production system and level of dirtiness, were the explanatory variables.

## Results

### Mastitis prevalence

The prevalence of subclinical mastitis across the herds was 51.8% (Table 1).

**TABLE 1:** Mastitis prevalence across milk collection centres.

MCC	Number of farms	Number of cows tested	Number of mastitis positive cows	% mastitis positive
Isangano	8	65	38	58.50
Kirebe	7	65	30	46.10
Nyagatare	8	65	33	50.70
Total	23	195	101	51.8*

MCC, milk collection centres.

*, 95% confidence limits were 44.5% – 59%.

### Mastitis risk factors

Out of the risk factors selected for inclusion in the logistic regression model, cowdirtiness, production system, breed, teat-end condition, and stage of lactation had significant (*P* < 0.01) associations with mastitis prevalence (Table 2). Age had no significant association (*P* > 0.05) with mastitis prevalence, whilst parity had a marginally significant association (*P* = 0.056) with mastitis prevalence (Table 2). Based on Cramer's V statistic value, teat-end condition had the strongest association with mastitis prevalence, followed by stage of lactation and then breed and cow dirtiness.

**TABLE 2:** Association between mastitis prevalence and mastitis risk factors.

Risk factor	Chi-square value	Degrees of freedom	Significance level (*P*-value)	Strength of association – Cramer's V
Cowdirtiness	12.061	2	0.0024	0.249
Production system	18.859	1	0.000016	0.31
Breed	12.087	2	0.0024	0.249
Teat-end condition	73.948	4	< 0.0001	0.616
Parity	3.314	2	0.0564	0.13
Stage of lactation	15.576	2	0.0004	0.283
Age	-	1	0.48	0.016

Mastitis prevalence numerically increased with an increase in teat-end damage and keratinisation (Table 3). In addition, severely damaged ulcerated teat-ends with scabs or open lesions were the most susceptible to mastitis. Mastitis prevalence increased with an increase in cow dirtiness (Table 4), whilst the extensive production systemhad a higher percentage prevalence of mastitis than the semi-intensive system (Table 5). Mastitis prevalence was higher for pure breeds (pure Friesian and Jersey), followed by cross-breeds and then local breed cows (Ankole) and Sahiwal (Table 6). It was also higher in late and early stages of lactation than in mid-lactation (Table 7).

**TABLE 3:** Prevalence of mastitis with regard to teat-end conditions in examined cows.

Teat-end condition	*N*	Mastitis		% mastitis positive
		Negative	Positive	
Smooth teat-end sphincter	38	32	6	15.80
Teat-end sphincter with raised smooth ring (no cracks)	56	42	14	25.00
Roughened teat-end sphincter Slight cracks, no redness	48	15	33	68.80
Inverted teat sphincter with many flower-like depressions/cracks	39	5	34	87.20
Teat-end severely damaged and ulcerative with scabs or open lesions	14	0	14	100
**Total**	**195**	**94**	**101**	**-**

**TABLE 4:** Mastitis prevalence in relation to cow dirtiness scores.

Cow dirtiness	*N*	Mastitis		% mastitis positive
		Negative	Positive	
Very dirty	4	1	3	75.00
Fairly or moderately dirty	129	52	77	59.70
Slightly dirty	62	41	21	33.90
**Total**	**195**	**94**	**101**	**-**

**TABLE 5:** Percentage mastitis prevalence in different production systems.

Production system	*N*	Mastitis		% mastitis positive
		Negative	Positive	
Semi-intensive system	85	56	29	34.10
Extensive system	110	38	72	65.50
**Total**	**195**	**94**	**101**	**-**

**TABLE 6:** Mastitis prevalence in different breeds.

Breed	Studied cows	Studied cows (%)	Mastitis		% mastitis positive
			Negative	Positive	
Exotic cow (pure breed)	35	17.90	9	26	74.30
Cross-breed	110	56.40	53	57	51.80
Ankole	50	25.60	32	18	36.00
**Total**	**195**	**100**	**94**	**101**	**-**

**TABLE 7:** Mastitis prevalence in different stages of lactation.

Stage of lactation	Examined cows	Examined cows (%)	Mastitis		% mastitis positive
			Negative	Positive	
1–2 months	67	34.40	29	38	56.70
3–6 months	82	42.1	52	30	36.60
7 months and over	46	23.6	13	33	71.70
**Total**	**195**	**100**	**94**	**101**	**-**

### Causal relationship of risk factors and mastitis prevalence

The logistic regression results showed that the risk factors teat-end condition, rearing system, cow dirtiness, stage of lactation and breed accounted for 62% of mastitis prevalence. Teat-end condition alone accounted for 29% out of the 62%, indicating that amongst the risk factors studied it was the most important determinant of mastitis. Teat-end condition was followed by rearing system, which accounted for 15.2% out of 62%, the third being breed with 14.96%, then cow dirtiness with 6.18%. Stage of lactation accounted for only 5.7% out of 62% of mastitis prevalence (Table 8).

**TABLE 8:** Causal relationships between mastitis risk factors and mastitis prevalence.

Source	Degrees of freedom	Significance level (*P*-value)	Figures
Corrected model	11	0	62
Intercept	1	0.034	-
Dirtiness	2	0.045	6.18
Rearing system	1	0	15.2
Stage of lactation	2	0.056	5.7
Teat-end condition	4	0	29
Breed	2	0.001	14.96

### Screening for mastitis-causative organisms

Coliforms accounted for the largest percentage of subclinical mastitis, followed jointly by environmental staphylococci and *Staph. aureus* (Table 9).

**TABLE 9:** Mastitis-causative organisms isolated from 16 cows with subclinical mastitis samples.

Causative micro-organism	No. of positives	% positive
Coliforms	14	87.5
Environmental Staphylococci	1	6.25
*Staph. aureus*	1	6.25
**Total**	**16**	**100**

## Discussion

The mastitis prevalence of 51.8% obtained in this study is lower than the 58.6% reported by Chatikobo (2010) in cows on a farm in the same District of Nyagatare using the Draminski^®^ Mastitis Detector. Surprisingly, this high prevalence and that obtained in this study were observed during the dry season, when mastitis prevalence is expected to be low. A possible reason for this high prevalence is the absence of mastitis prevention and control programmes, including post-milking teat dipping with disinfectant and antibiotic dry cow therapy on most farms.

The prevalence of mastitis found in this study is lower than that in the studies of Janzekovic *et al.* (2009) in Slovenia, Muhammad *et al.* (2011) in Pakistan, and Siddiquee *et al.* (2013) in Bangladesh, all of which used the Draminski^®^ test and reported prevalence values of 80%, 65.2% and 55.1% respectively. However, prevalence of mastitis in this study was higher than that reported in other studies that used the Draminski^®^ test: Hassan, Samarasinghe and Lopez-Benavides (2007) reported a prevalence rate of 39% in New Zealand, and Yogesh *et al.* (2014) reported a prevalence of 46.4% in India.

Whilst the Draminski^®^ test based on electrical conductivity is regarded as a low-precision cow-side mastitis screening test, Anil *et al.* (2014) reported compatibility between the results of somatic cell counts and Draminski^®^ tests, in which the somatic cell count detected 64.4% of subclinical mastitis in cows whilst the Draminski^®^ test detected 59%.

As stated by Levesque (2004), herd mastitis prevalence of 40% or over must sound an alarm to the producer. It is clear that the high mastitis prevalence in Nyagatare is a serious problem that not only reduces milk production but adversely affects the quality of milk and leads to economic losses.

This study revealed an association of mastitis with teat-end condition, cow dirtiness, breed, production system and stage of lactation, that are largely related to management and rearing environment. According to Radostitis, Arundel and Clive (2000) mastitis is a complicated problem, associated with almost every conceivable factor of management and the environment. Teat-end condition has also been mentioned by Biffa *et al.* (2005) and Mekibib *et al.* (2009) as a factor influencing mastitis prevalence. Extra teats predispose to mastitis and may interfere with milking. Inverted teat-ends are least resistant to infection compared to pointed (rounded) teat-ends (Katunguka-Rwakishaya & Ndikuwera 2008). Cows with leaky sphincters may also be more susceptible to infection (Katunguka-Rwakishaya & Ndikuwera 2008). However, it is acknowledged that this study was conducted in an area with savannah vegetation and predominantly extensive production systems, which may expose cows toteat-enddamage and hence predispose the teats to mastitis infections.

With regard to the association of cow dirtiness with mastitis prevalence, the results are in agreement with the findings of Rahman *et al.* (2009) in Bangladesh. However, according to the National Mastitis Council (2003) the level of dirtiness is subjective – what appears ‘dirty’ to one individual may appear ‘normal’ to another. As a result the discussion of what is ‘clean’ is often driven by opinion rather than fact. However, in this study the authors attempted to objectively classify the level of cow dirtiness using a scale based largely on the part of the body covered by dirt and the extent of coverage, as described by Chiappini *et al.* (1994).

In agreement with the results of this study, Siddiquee *et al.* (2013), using the Draminski test, reported that cows with 75% Holstein Friesian genotype experienced a higher prevalence of subclinical mastitis (63.0%) than other genotypes.

Results from elsewhere corroborate the association of production systems with mastitis prevalence found in this study. In this regard, Abera *et al.* (2010), Biffa *et al.* (2005) in Ethiopia and Kivaria, Noordhuizen and Msami (2006) in Tanzania found that mastitis prevalence was higher in extensive than in intensive production systems. Oliver *et al.* (2004) in Tennessee in the United States of America found that mastitis was a problem related to housing systems.

As found in this study, Abera *et al.* (2010) in Ethiopia, Fadlelmoula *et al.* (2007) in Germany and Girma (2007) in Ethiopia all reported that stage of lactation is an important risk factor. According to Kivaria *et al.* (2006), about 60% – 70% of these cases actually start at the end of the previous dry period or during calving. However, some of these early lactation infections are spontaneously cured in a few days.

Although this study did not show age and parity as significant causes of mastitis in cows, Siddiquee *et al.* (2013) reported an increase in subclinical mastitis prevalence with increases in age and parity. According to Levesque (2004) mastitis risk factors vary from farm to farm, and their assessment is considered to be the first step in mastitis control programme formulation adapted to the specific farm situation. It is acknowledged that record-keeping amongst farmers in this study was weak, hence data on parity and age may not be accurate, particularly where most of the cows were bought into the herds.

The high percentage of mastitis caused by coliform bacteria indicates contamination from soil and faecal matter. According to Mellenberger and Roth (2009) coliform bacteria are normal inhabitants of soil and the intestines of cows. They accumulate and multiply in manure, polluted water, dirt and contaminated bedding. According to Kivaria *et al.* (2006) coliforms invade the udder through the teat sphincter when teat-ends come into contact with an environmental site that is contaminated with coliform organisms.

Thus, based on the results found in this research, it is likely that the high prevalence of mastitis in Nyagatare herds is a consequence of poor hygiene and teat-end condition. Clearly most mastitis cases were largely of environmental origin.

## Conclusion

The overall prevalence of mastitis at farm level in Nyagatare District was 51.8%. This indicates that mastitis is a serious problem across herds in this district. The main risk factors associated with the prevalence of mastitis, in order of importance, were teat-end condition, production system, cow dirtiness, stage of lactation and breed. Mastitis infections were largely caused by coliform bacteria (87.5%), *Staph. aureus* (6.25%) and environmental staphylococci (6.25%). These micro-organisms are associated with poor hygiene and contamination of udders and milking equipment with soil and faeces.

It is recommended that dairy farmers in Nyagatare District introduce mastitis control programmes, particularly teat dipping, dry cow therapy and effective treatment of clinical mastitis, to reduce the high prevalence of mastitis in their herds.
